# Lymphangiectasis of lower limb

**Published:** 2009

**Authors:** S. B. Gogia

**Affiliations:** Sanwari Bai Surgical Centre, 28/31 Old Rajinder Nagar, New Delhi - 110 060, India

The authors deserve to be congratulated for their excellent management and overall good result in a very challenging case. However, there is an issue regarding the diagnosis of the patient. Lymph oedema seems to be a neglected problem with overall poor outcomes and any perceived success brings about a sense of bewilderment. The flow of arguments to our minds suggests that the authors have been trying to justify the fact that the diagnosis was not lymph oedema because they got a good outcome!!!

To quote -

“Final diagnosis was established by lymphangiography and lymphoscintigraphy, which revealed marked dilatation of lymphatic channels in leg and inguinal region.”

The above is a hallmark of filariasis and occurs early in patients (this patient was 24 years of age), a stage which Pani *et al.*[1] called reversible oedema. Added to that, the patient was seen in a lymph oedema clinic being run in an endemic area (Eastern UP) with all clinical features, suggestive of lymph oedema. So to my mind, this patient had lymph oedema. Admittedly, such features are found more in Brugia Malayi (found in Kerala) where hydrocoel is not a feature (no mention in the data presented; so presumably, it was not present).

We find no mention of a test for filariasis. Although night blood smear examination is frequently false negative, serum filarial antigen testing has been in vogue since many years and should have been done. The authors themselves state that classic features of lymphangiectasis – in the form of visceral and bone involvement were not present. We would rather take the argument upside down i.e. if it was lymphangiectasis, the stated good results may not have been present.

We have attempted to treat both conditions and inevitably found better results in lymph oedema, especially if it is of filarial origin rather than any other cause. Of late, however, our results in post mastectomy lymph oedema are improving with CDT.[2] Results are worse in primary and other congenital causes of lymph oedema, wherever there is a paucity of lymphatic vessels. Surgery can remove the diseased tissue partly; recurrence is more dependent on the aftercare, notable prevention of infection.[3]

However, the excellent management and results do need to be show cased. We have to re- emphasise that with current management techniques, lymphoedema, especially of filarial origin, is treatable and occasionally curable. Surgery should, however, be used as an adjunct for the more advanced cases like the present one rather than stand alone therapy.
